# The Australian Bogong Moth *Agrotis infusa*: A Long-Distance Nocturnal Navigator

**DOI:** 10.3389/fnbeh.2016.00077

**Published:** 2016-04-21

**Authors:** Eric Warrant, Barrie Frost, Ken Green, Henrik Mouritsen, David Dreyer, Andrea Adden, Kristina Brauburger, Stanley Heinze

**Affiliations:** ^1^Lund Vision Group, Department of Biology, University of Lund Lund, Sweden; ^2^Department of Psychology, Queens University Kingston, ON, Canada; ^3^New South Wales National Parks and Wildlife Service Jindabyne, NSW, Australia; ^4^Institute for Biology and Environmental Sciences, University of Oldenburg Oldenburg, Germany

**Keywords:** Bogong moth, *Agrotis infusa*, insect, migration, navigation, estivation, vision, magnetoreception

## Abstract

The nocturnal Bogong moth (*Agrotis infusa*) is an iconic and well-known Australian insect that is also a remarkable nocturnal navigator. Like the Monarch butterflies of North America, Bogong moths make a yearly migration over enormous distances, from southern Queensland, western and northwestern New South Wales (NSW) and western Victoria, to the alpine regions of NSW and Victoria. After emerging from their pupae in early spring, adult Bogong moths embark on a long nocturnal journey towards the Australian Alps, a journey that can take many days or even weeks and cover over 1000 km. Once in the Alps (from the end of September), Bogong moths seek out the shelter of selected and isolated high ridge-top caves and rock crevices (typically at elevations above 1800 m). In hundreds of thousands, moths line the interior walls of these cool alpine caves where they “hibernate” over the summer months (referred to as “estivation”). Towards the end of the summer (February and March), the same individuals that arrived months earlier leave the caves and begin their long return trip to their breeding grounds. Once there, moths mate, lay eggs and die. The moths that hatch in the following spring then repeat the migratory cycle afresh. Despite having had no previous experience of the migratory route, these moths find their way to the Alps and locate their estivation caves that are dotted along the high alpine ridges of southeastern Australia. How naïve moths manage this remarkable migratory feat still remains a mystery, although there are many potential sensory cues along the migratory route that moths might rely on during their journey, including visual, olfactory, mechanical and magnetic cues. Here we review our current knowledge of the Bogong moth, including its natural history, its ecology, its cultural importance to the Australian Aborigines and what we understand about the sensory basis of its long-distance nocturnal migration. From this analysis it becomes clear that the Bogong moth represents a new and very promising model organism for understanding the sensory basis of nocturnal migration in insects.

## Introduction

**Figure 1:**
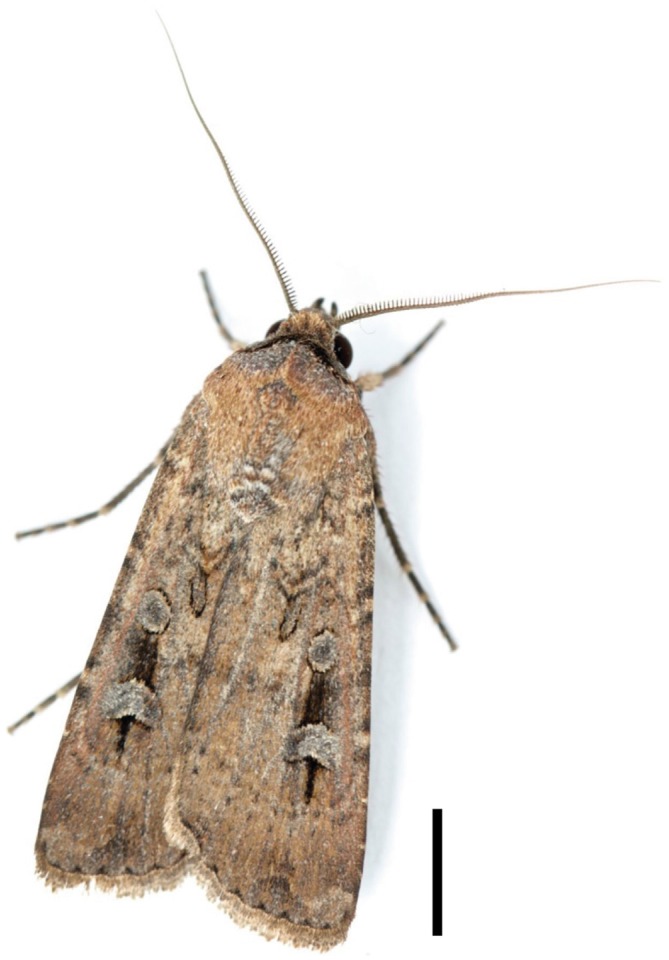
**The Australian Bogong moth *Agrotis infusa* (Boisduval, 1832).** Scale bar = 5 mm. Reproduced with the kind permission of the photographer; Ajay Narendra, Macquarie University, Australia.

**Figure 2:**
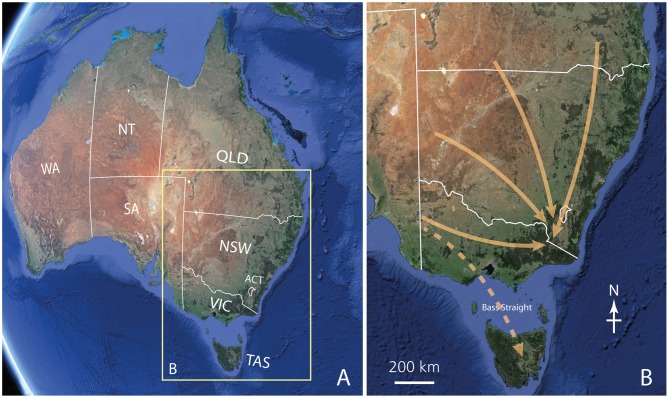
**The migratory routes of Bogong moths. (A)** A map of Australia showing the Australian States and Territories (WA, Western Australia; SA, South Australia; NT, Northern Territory; QLD, Queensland; NSW, New South Wales; ACT, Australian Capital Territory (the location of the national capital Canberra), VIC, Victoria; TAS, Tasmania). The yellow box shows the region of Australia shown in **(B). (B)** The likely migratory routes of Bogong moths during the spring migration (the autumn migration occurs in the reverse direction). *Arrows* show the migration of moths to the alpine regions of NSW, the ACT and Victoria from southern Queensland, western and northwestern NSW and western Victoria. Some moths, blown from western Victoria by northwesterly prefrontal winds, even cross Bass Straight to arrive in Tasmania (where they either breed or estivate, returning to Victoria during what appears to be a reverse migration in autumn). Because we are currently unsure whether this is a true migration or an accidental displacement by winds, the Tasmanian arrow is shown as *dashed*. Images taken from Google Earth.

**Figure 3:**
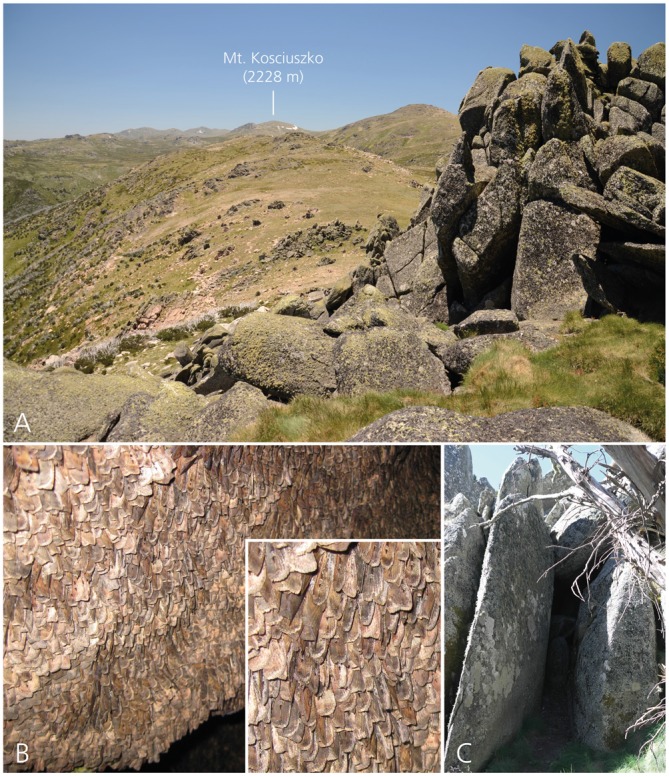
**Bogong moth estivation. (A)** The typical terrain of the Main Range of the Snowy Mountains, seen in early January from the summit of South Ramshead (2052 m), looking north towards Mt Kosciuszko, Australia’s highest mountain (2228 m). The landscape of treeless grassy meadows is dotted with complex rock tors, periglacial boulder fields and boulder streams, some of which provide estivation sites for Bogong moths. Photograph: Stanley Heinze. **(B)** Bogong moths estivating in a cave on South Ramshead (inset: close-up). Photograph taken by Eric Warrant in late December. **(C)** A typical cave entrance within a rocky tor on the summit of Mt Gingera (1857 m) in the Brindabella Ranges on the border between NSW and the ACT. Mt Gingera is the northernmost known site for Bogong moth estivation. Photograph: Eric Warrant.

Every spring, newly eclosed Bogong moths *Agrotis infusa* (Figure 1)—modest-looking brown nocturnal moths of the family Noctuidae—embark on a remarkable long-distance migration of up to 1000 km towards the high alpine areas of southeastern Australia (Figure 2). Flying from the dry plains of southern Queensland, western and northwestern New South Wales (NSW) and western Victoria, they seek out the shelter of cool mountain caves and rock crevices dotted across the alpine landscape, gradually congregating there in their billions (Figure 3; Green, 2010a). Here they stay in a dormant torpid state (known as “estivation”) for up to 4 months, neatly tiling the cool rock walls of the caves (Figure 3B) until the beginning of the following autumn—an astonishing 17,000 of them per square metre (Common, 1954). Then, awakened from their long dormancy, these moths leave their caves and return to where they came from, making long journeys back to their breeding grounds. Once there they mate, lay their eggs and die, and the Bogong moth life cycle starts afresh.

Not surprisingly, this remarkable natural phenomenon has not gone unnoticed by human observers. For tens of thousands of years, Australia’s first inhabitants, the Aborigines, knew of the vast numbers of moths resting in the mountains above them, and tribes originating from both sides of the range ascended to high alpine meadows each summer to feast on the rich source of fat and protein provided by these insects and to attend to intertribal business (Flood, 1980, 1996). In more recent times, the bright light pollution of nearby Canberra—Australia’s national capital—has occasionally diverted Bogong moths from their normal migratory route, leading to plagues of moths in public buildings, where they block air ducts, short out electrical circuits and ignite mild panic among those less tolerant of the vagaries of the natural world.

The details of the Bogong moth migration were not understood until the early 1950’s, when Dr. Ian Common, Australia’s foremost moth specialist at the Commonwealth Scientific and Industrial Research Organisation (CSIRO) in Canberra, undertook a thorough study of Bogong moth estivation sites in the nearby Brindabella Ranges, a string of high mountains running along the western edge of the Australian Capital Territory (ACT; Common, 1952, 1954). Since that time, further studies using a variety of methods—including vertical radar (Drake et al., 1981; Drake and Farrow, 1985), long-term light trapping (Gregg et al., 1993, 1994) and careful observation (Green, 2006, 2008, 2010b, 2011)—have confirmed and extended Common’s work and given us a reasonably clear picture of the migration and natural history of the Bogong moth. What is apparent from these studies is that the migration of the Bogong moth has much in common with that of the well known and much better studied Monarch butterfly (*Danaus plexippus*) from North America. In autumn, single individuals of this day-active butterfly migrate up to 4000 km from southern Canada and the northern United States to specific overwintering sites in Mexico, with the return migration occurring over several generations during the following spring (Urquhart, 1987). In contrast, the Bogong moth is strictly nocturnal and the same individual makes both the forward and reverse journeys.

Even though the migration of the Australian Bogong moth is less well understood than that of its more colorful day-active North American counterpart, it is no less spectacular. Like the Monarch butterfly, naïve Bogong moths begin an immense journey to a place they have never previously visited, potentially using a variety of sensory cues to follow a genetically inherited preprogramed route and to stop at the correct destination. In the case of the Bogong moth, this must then be reversed months later to return to their breeding grounds. But unlike the Monarch butterfly, which predominantly uses reliable celestial visual cues (the sun) as a compass to steer its course in the desired migratory direction (Mouritsen and Frost, 2002; Heinze and Reppert, 2011), the Bogong moth may need to navigate at night using visual cues that are far dimmer and much less reliable. As we will argue below, long-distance visual navigation at night is far from trivial (Warrant and Dacke, 2010, 2011, 2016), and other sensory cues (such as the Earth’s magnetic field) may provide a more reliable compass.

The well-defined migration of the Australian Bogong moth thus provides an outstanding system for studying the ecological, sensory and neural basis of long-distance nocturnal navigation (Heinze and Warrant, 2016). In this review we will first describe the moth’s natural history and its cultural importance to the Australian Aborigines. The final part of the review, deals with what we currently understand about the moth’s migratory behavior, both in terms of its biogeography and its sensory basis. Far from being yet another dull brown moth, the Bogong moth emerges as a most extraordinary insect.

## The Aboriginal Cultural Relationship with the Bogong Moth

**Figure 4:**
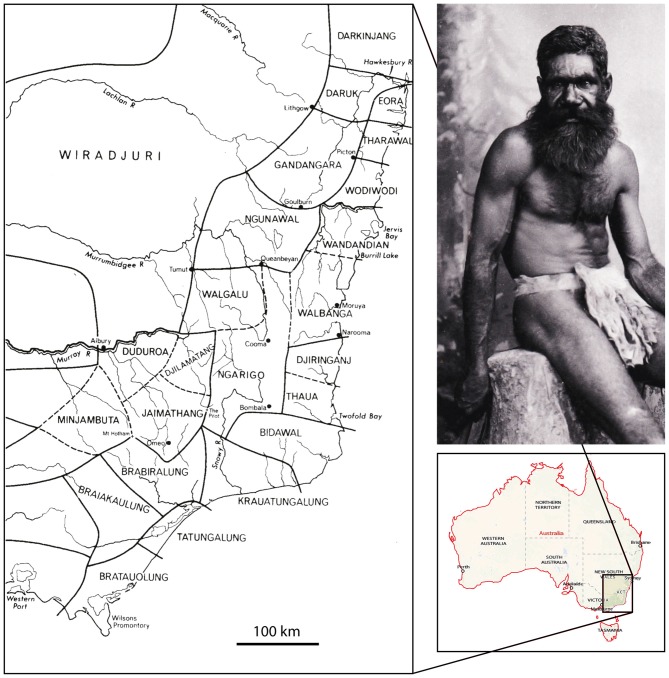
**The traditional Aboriginal tribal boundaries of southeastern Australia, and a 19th century portrait of an Aboriginal man from the Monaro district of the Snowy Mountains wearing the apron-like bridda bridda.** The map is a reproduction of Tindale’s 1974 map of indigenous group boundaries existing at the time of first European settlement in Australia (Tindale, 1974). It is not intended to represent contemporary relationships to land. © Tony Tindale and Beryl George, 1974. Portrait: Photo no. 1304 by Henry King (1855–1923), from the Tyrrell Collection (7903 glass plate negatives from the studios of Henry King and Charles Kerry (1858–1928), held at the Powerhouse Museum, Sydney, and available through the Commons on Flickr).

Perhaps one of the major reasons for the iconic status of the Bogong moth for Australians is the critical role they played as a major food source for Aboriginal tribes in southeastern Australia and particularly those whose home territories were within or adjacent to the alpine regions of the Southern Tablelands. In her comprehensive book on the subject, aptly named *The Moth Hunters*, Josephine Flood (1980) documents considerable evidence, from both traditional Aboriginal oral sources and early European writings, outlining how several Aboriginal tribes converged on the high peaks of mountain ranges in the ACT and in the Snowy Mountains of NSW to harvest and then feast on Bogong moths from November to February, with Aboriginal numbers reaching a peak around the end of December. One account estimated that gatherings could be as large as 500–700 Aborigines belonging to different friendly tribes. However, these journeys to the heart of the Bogong moth estivation areas were not only intended for collecting and feasting on moths. They also clearly served a deeper cultural purpose—intertribal meetings, initiation rites and corroborees, marriages, trade facilitation, and fostered mutual understanding and friendship, were all important priorities. Although most of this activity was centered around Mt Kosciuszko (Figure 3) and the Bogong Peaks (in NSW, about 50 km west of the ACT), Flood (1980, 1996) documents that Bogong moth tribal gatherings also occurred in the Brindabella Ranges (that run along the western borders of the ACT) and in the Tinderry Mountains (in NSW, immediately to the east of the ACT, just beyond the border). Aborigines also gathered further south in the Victorian Alps. The map shown in Figure 4 (after Tindale, 1974; from Flood, 1980) illustrates the rough territorial boundaries of various Aboriginal clans or tribes. As in other indigenous groups, their affiliations were essentially forged by kinship and commonalities in language, with adjacent tribes—if not speaking a common language or dialect—being sufficiently multilingual to enable communication with their neighbors. Since a fairly widespread custom (or Aboriginal law) required marriage outside the clan, these intertribal meetings, occasioned by the Bogong moth harvest, provided an excellent opportunity for facilitating these marriages.

Although there are no historical records (or oral history accounts) of how the Aborigines originally located the Bogong estivation sites, it is quite possible that they used the aggregation of ravens in the vicinity of the Bogong moth caves as an indication of nearby estivation sites. In his book about an ancient Aboriginal route from the coast to the mountains, *On Track: Searching out the Bundian Way*, Blay (2015) describes how, at the end of the summer, Little ravens (*Corvus mellori*, or Arabul as they were called in an Aboriginal dialect: Blay, 2015) congregated in their hundreds to feast on the Bogong moths that were leaving their estivation sites to begin their autumnal migration back to their breeding grounds. Whether or not the ravens also signaled the springtime arrival of the Bogong moths into the high country is unclear, but in an interesting account of the Australian Broadcasting Corporation’s filming of a documentary about the Bundian Way, John Houstin (2015) writes that after a rather disappointing search for Bogong moths the team witnessed what John Blay described as “Hundreds of thousands (sic) of black ravens suddenly (appearing) out of the crevices in the granite where they’d been hunting the moths”.

The traditional harvesting of Bogong moths from the their estivation sites was carried out only by Aboriginal men, who according to Flood (1980, 1996), used sticks to scrape them off the vertical walls of the fissures in the granite rocks (see Figure 3), and collected them in a bark dish (called a “coolamon”), or in finely woven nets made by the women from *Pimelia* or *Kurrajong* fiber gleaned specifically for this purpose. A European account of the Bogong moth harvest by Helms (1895), based on his conversations with a settler who had lived in the area between 1850 and 1890, described how the Aboriginal men sometimes used a burning or smoldering bush which they took into the caves and clefts to stun the moths temporarily with smoke, causing them to fall to the floor where an outstretched kangaroo skin or a fine fiber net aided their collection.

Once harvested, Bogong moths were prepared for eating by roasting them carefully in hot ashes. Several accounts refer to the Bogong moths as a “relished” food much sought after by the Aborigines who feasted on them for many weeks. Early European settlers who ate them described them as being “exceedingly nice”, tasting sweet and walnut-like. Not only were the Bogong moths tasty, it appears they were also highly nutritious, having approximately 60% fat by dry weight (Common, 1954) and 27% protein (Flood, 1996). Helms (1895, as cited in Flood, 1980) described the physical condition of the Aborigines as generally improved after their time in the mountains, with their “skin being glossy and most of them quite fat”.

## The Lifecycle of the Bogong Moth

**Figure 5:**
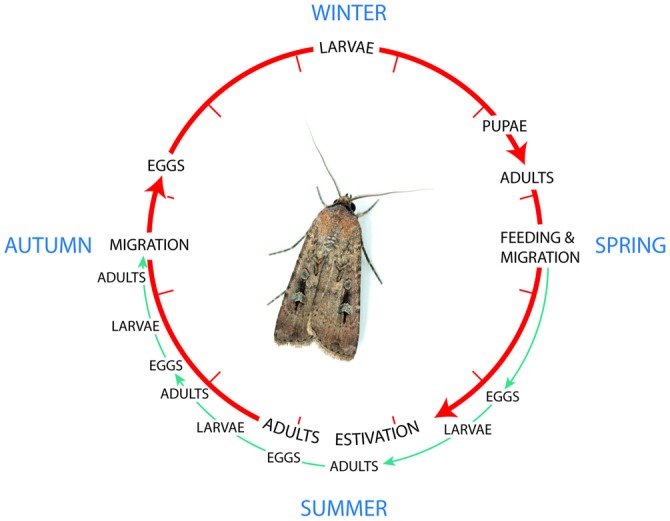
**The lifecycle of the Bogong moth, a multivoltine species with the potential for three overlapping generations in favorable conditions where there is access to larval food plants *green pathway*.** However, in most Bogong moth breeding areas conditions do not remain favorable, and moths from such areas undergo a spring migration to escape the unsustainable conditions (*red pathway*). Re-drawn from Common (1954). Photo of the Bogong moth courtesy of Ajay Narendra, Macquarie University, Australia.

The Bogong moth *Agrotis infusa* is a multivoltine species (i.e., having the potential for several broods within a season) with the potential for three overlapping generations in favorable conditions where there is access to larval food plants (annual dicotyledons) throughout the spring and summer (Figure 5, Common, 1954; McQuillan et al., 2007). However, in most Bogong moth breeding areas conditions do not remain favorable, with larval food plants, abundant during the winter, becoming scarce later in the spring and over the summer unpalatable perennial grasses predominate (Common, 1954). Bogong moths from such areas undergo a spring migration to escape the unsustainable conditions.

Nonetheless, there are also isolated populations of Bogong moths living in favorable areas that are non-migratory. One such population is clearly found in the ACT and southeastern NSW. Ian Common’s permanent light-trap on Black Mountain, within the metropolitan area of Canberra in the ACT, caught non-migratory adults (distinguished by hind-wing color—see below) from May to early August, while the same light-trap recorded migratory adults (flying southwards towards the Alps) only from late September to early December. Another population is found in Western Australia (WA) south of Perth. Although this population contains moths with both hind-wing colors it is possibly non-migratory due to the dearth of alpine areas suitable for estivation (Ted Edwards CSIRO, personal communication).

### Eggs

A female Bogong moth can lay up to 2000 eggs (Common, 1981) which are dome–shaped, vertically ridged, 0.7 mm in diameter and 0.4 mm in height (McQuillan et al., 2007). They are laid in clusters in the soil or, if soil conditions are not suitable, on the foliage and stems of plants, including trees (Hill, 2007; McQuillan et al., 2007). Incubation time for eggs in the laboratory varies with temperature, ranging from 4 to 5 days at 24°C to 7 days at 18°C with no hatching at 4.5°C (Common, 1954). Although migrating Bogong moths are sexually immature, adults fed on a sugary food can lay eggs with the pre-oviposition period of moths in spring, ranging from 24 to 66 days (Common, 1954).

### Larvae

The larvae of the Bogong moth develop through six instars. Larger caterpillars cut the stems of low-growing plants at ground level for food (Common, 1981; McQuillan et al., 2007)—hence the common name “cutworm moth” for moths of the genus *Agrotis* (and several other noctuid genera). The frequently pink-tinged larvae grow to a maximum length of about 50 mm (McQuillan et al., 2007). No larval diapause occurs and larvae grow slowly in winter reaching about the third instar in June, with accelerated growth in spring to reach the final instar in late August and early September (Common, 1954, 1981). In Tasmania, where a proportion of the migratory spring generation may breed (due to the displacement of Bogong moths across Bass Straight by prefrontal winds from the Australian mainland, see below), larvae occur from October to March and reach the final (sixth) instar by December-January (Hill, 2007; McQuillan et al., 2007).

Bogong moth larvae are found within large areas of Australia, south of the Tropic of Capricorn including Tasmania (Common, 1990). They extend from coastal regions of NSW in favorable years, westward to Spencers Gulf in South Australia (SA; Common, 1954). Based on sightings of adults, Bogong moth larvae may even extend further west towards WA. Adult Bogong moths have been recorded as far west as Perth, WA (Ken Green, personal observations).

**Figure 6:**
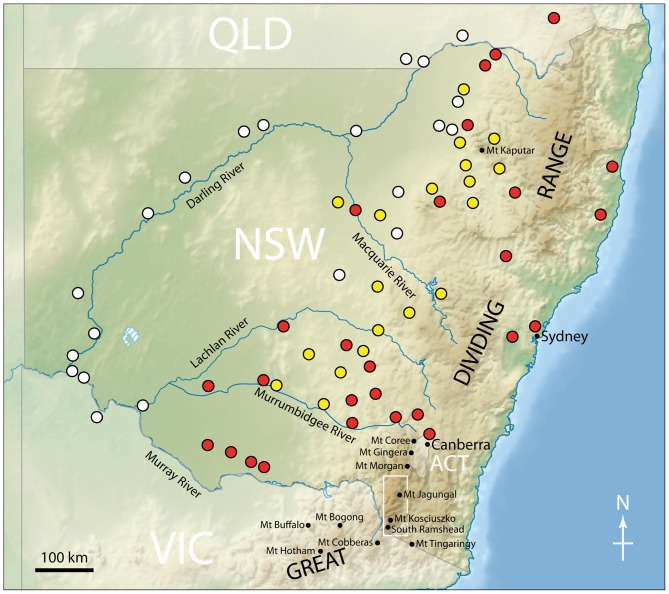
**Larval collection locations in southeastern Australia.** Collections were made by Froggatt (1900, *yellow circles*), Common (1954, *red circles*) and Green (2008, *white circles*). Also shown are the names of the major rivers in NSW, and the locations of various mountain peaks mentioned in the text. The *white box* indicates the region of the Snowy Mountains shown in Figure 9. Redrawn from Green (2008).

Larvae have been found in alluvial soils along the Darling River in western and northwestern NSW at elevations of 50–275 m (Green, 2008; Figure 6). Larvae occurred across a variety of land types, including cropland (cotton, wheat and vegetables), pasture, orchards (apple and orange) and vineyards, all of which had broadleaved weeds, particularly clover. In large areas further east in NSW they were most commonly found in dense stands of the medick *Medicago* spp. (Common, 1954). Larval collections for arsenic studies were conducted from late July to late August 2001 and densities ranged from 0 to 4.0 larvae m^−2^, with the highest density associated with cutworm larval damage. The larvae prefer broad-leaved plants rather than grasses and cereal (Common, 1981), although at times they damage pasture and crops such as wheat, barley, linseed, lucerne, peas, potatoes, cabbages, cauliflower and silver beet (Common, 1990). Although their main food source before European settlement remains unknown, the only native species on which damage has been reported is saltbush (Common, 1954).

Summer populations of larvae can be found in a number of favorable areas. No larvae have been found in the vicinity of estivation sites but occasional larvae resulting from the spring migration have been found in summer in gardens in Canberra (Common, 1954). Moths also breed throughout summer in the Adelaide area (Rawat, 1958) and in Tasmania (Hill, 2007). There is no evidence of a permanent larval population in Tasmania—instead, populations arising each year in Tasmania are the result of repeat spring migrations from the Australian mainland (Hill, 2007), most likely due to accidental displacements across Bass Straight by prefrontal northwesterly winds (Drake et al., 1981; Drake and Farrow, 1985). Part of this migrating population estivates, although some moths breed, laying eggs in agricultural districts where food plants are emerging, predominantly in northwestern Tasmania (Hill, 2007). Fully-grown larvae typically occur from December to January (McQuillan et al., 2007), and the crops from which summer larvae have been collected include sweet corn, potato, squash, poppy, rye corn, barley, green bean, carrot, swede, Chinese cabbage, sugarbeet, silverbeet, hops, lettuce and clover. They were also found among dock and mallow, and in domestic lawns (Hill, 2007).

### Pupae

The pupa of the Bogong moth is a shiny, brown capsule 20 mm long (McQuillan et al., 2007). Pupation occurs at a soil depth of about 20–30 mm (Common, 1981), although in Tasmania it occurs at a depth of 20–150 mm (McQuillan et al., 2007). At 24°C the time taken to develop from egg to adult is about 7 weeks (Common, 1954). Pupation can pass in as little as 3 weeks, but in Tasmania most pupating moths do not emerge until February (McQuillan et al., 2007), just in time to join the autumnal migration back to the mainland.

### Adults

The Bogong moth, with a wingspan of 40–50 mm (Common, 1981; our own measurements), is smaller than most people think. The head-body length is approximately 20–25 mm, 25–35 mm when measured to the wingtip (McQuillan et al., 2007; our own measurements). It is easily identified from similar dark brown moths by the two conspicuous spots on each wing. Moths from a cave on South Ramshead (a rocky peak in the Snowy Mountains) weighed on average 0.33 g with an oven-dry mass of 0.16 g (Green, 2011). On arrival in the mountains their abdomen contains 66% fat (by dry weight) in males and 57% in females (Common, 1954).

**Figure 7:**
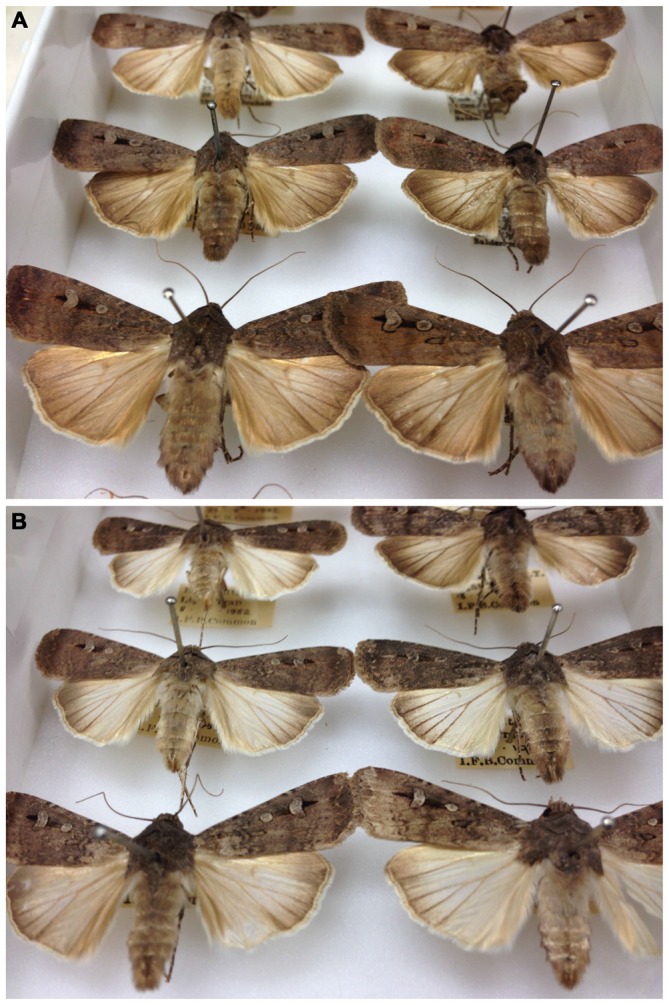
**Migratory (A) and non-migratory (B) morphs of the Bogong moth.** Note the paler hindwings of the non-migratory morph. Photographs taken in the Australian National Insect Collection, Canberra (Photos: Eric Warrant).

Bogong moth adults come as two morphs, one apparently a migratory morph, the other non-migratory (Figure 7). The non-migratory morph has whitish hind wings and occurs during the winter months from May. By September only occasional worn specimens of the non-migratory morph can be found (Common, 1954). This morph does not migrate *en masse* and is not gregarious (Common, 1954). The migratory morph, in contrast, has brown hind wings and appears in September, and is the only morph that is caught in spring and autumn (Common, 1954).

## The Bogong Moth Migration

As mentioned earlier, Bogong moths make a yearly migration in the spring, taking off from their breeding grounds in southern Queensland, and western and northwestern NSW, and flying great distances over many nights (or possibly even weeks) to arrive in the alpine regions of NSW, the ACT and Victoria (Figure 2). Some moths from western NSW, caught by prefrontal winds, may be carried across the sea as far as Tasmania, or even as far as New Zealand, a further 2000 km away (where less is known of their fate). At the end of the summer, the same individuals perform the reverse migration, returning to their breeding grounds.

### What Triggers Migration?

The spring migration begins in September (Common, 1954). The ultimate cause of this migration is the unsuitability of perennial summer grasses for supporting further generations of larvae (Common, 1954), and increasing temperatures may also act as a trigger (Common in Flood, 1980). However, the proximate trigger to migration is still unknown. There is no significant correlation between the time of arrival of Bogong moths in the mountains and effective late winter rainfall (rainfall over evaporation) in their breeding areas in southern Queensland, and western and northwestern NSW (Green, 2010a). A possible trigger for migration is the large and systematic changes in day length that occur during spring and autumn, a cue that could be tracked using a circadian clock, as proposed for Monarch butterflies (Reppert, 2006; Reppert et al., 2016). In migratory birds, cues that stimulate the onset of migration are also under the control of a biological clock, in this case a circannual clock (Gwinner, 1996).

### The Migratory Journey

#### Food

During the spring migration Bogong moths feed *en route* (McCarthy, 1945; Common, 1954). Bogong moths passing through the Grampians in western Victoria feed on early spring flowers from genera such as *Prostanthera*, *Lecopogon* and *Correa* (Cockburn, 1981). In NSW they also feed on *Epacris*, *Grevillea* and *Eucalyptus* (Common, 1954). Although normally nocturnal they can also feed during the day (Common, 1981), and they continue to feed right up to their arrival in the mountains where they may still feed for a while before entering estivation. In December the Snow gum *Eucalyptus niphophila* flowers from lower elevations in subalpine woodland right up to the treeline. Moths caught at this time of year, close to an estivation site, smelled strongly of honey (Ken Green, personal observations). In a study of pollination biology above the treeline in the Snowy Mountains, Inouye and Pyke (1988) found that Bogong moths visited and carried pollen of only one species, the white flowering Swamp heath *Epacris paludosa*, a species that in the year studied peaked in flowering before the end of December. Once moths move into their estivation sites they no longer feed (Common, 1954, 1981).

Food availability for the autumnal (return) migration might be expected to be lower than in the spring, but unlike army cutworm moths (*Euxoa auxiliaris*) which rely on fat stored over the summer for migration (Kevan and Kendall, 1997), Bogong moths are known to feed *en route* during the return migration, with feeding recorded on honeydew produced by lerp-forming bugs (Green, 2006).

#### Temporary Shelters

The spring migration across the lowlands can be quick, with some moths calculated as flying a few hundred kilometers in a single night (from vertical radar measurements of flight speeds: Drake and Farrow, 1985). However, once they arrive at the foot of the mountains the migratory pace declines and they ascend the mountains quite slowly. Because they start to travel slowly once in the mountains, and because streams of moths are converging from a number of source areas into more limited areas, high densities of moths accumulate and temporary clusters begin forming in suitable sites.

During the first long-distance phase of their migration, Bogong moths do not appear to use caves as temporary (or permanent) shelters, even when available and in suitably high-elevation locations (see Figure 6 for the locations of some peaks mentioned below). For Bogong moths traveling southwards from Queensland to the NSW alpine areas, Gregg et al. (1993, 1994) found no evidence for their aggregation in suitable high elevation estivation sites in northern NSW along the Great Dividing Range (the continuous range of mountains running down the entire east coast of Australia), either on its eastern side (Point Lookout, 1560 m) or its western side (Mt Dowe, 1460 m). Instead, these authors suggested that a less conspicuous form of the estivation strategy might occur locally in the humid pastoral zone at high elevations. However, searches during the spring migration of 2015 on Mt Kaputar—a mountain with elevation 1510 m on the migratory route in northern NSW—found caves sheltering Granny’s Cloak moths *Speiredonia spectans* (Erebidae), but no evidence of Bogong moths.

However, as moths approach the alpine regions they sometimes use caves as temporary camps at lower elevations, especially when the higher estivation caves are still buried in snow, only moving to caves at higher elevations as the season progresses (Common, 1981). Lower elevation temporary camps also include hollows in trees or logs as well as roadside cuttings (Common, 1954). As moths move higher in elevation they use periglacial boulder fields, tree hollows and stumps as temporary accommodation.

Mt Gingera (35°35′S 148°47′E, 1857 m), on the NSW/ACT border, is the most northerly known permanent summer estivation site (Common, 1981) but regular temporary camps exist somewhat further north at Mt Coree (35°18′S 148°49′E, 1421 m), and on higher western outliers of the Snowy Mountains, such as the Dargals Range (see Figure 9; Green, 2010b). Moths have also been found in a split rock “cave” at an elevation of 1020 m in the southern ACT (Winston-Gregson in Flood, 1980, p. 174). They can also aggregate during the spring migration in buildings (Common, 1981). Moths are attracted by the lights of buildings, such as those of Parliament House in Canberra, and seek darker places as daylight arrives. On the following night the lights are on again, and the moths become trapped by them. So clustering in such places is due to an inability to escape from a light constantly on, and is thus unnatural (Ted Edwards, personal communication).

#### Predators *en route*

During the migration, Bogong moths are preyed upon by a variety of birds, mammals and even fish. Brown trout (*Salmo trutta*) have been recorded with stomachs full of the remains of Bogong moths in lower elevation rivers below estivation sites, during both the spring and autumn migration periods (McKeown, 1955). Of the mammals, two species of bat (*Chalinolobus gouldii* and *Nyctophilus geoffroyi*) have been recorded taking Bogong moths during the autumn migration in NSW (Vestjens and Hall, 1977), and the Smoky mouse (*Pseudomys fumeus*) has been recorded as a predator in the Grampians (in western Victoria), during both the spring and autumn migrations (Cockburn, 1981).

#### Arrival in, and Departure from, the Mountains

The mean day of arrival of the first Bogong moths in the vicinity of the estivation sites was September 24th, plus or minus 1 week, from 1979 to 2009 (Green, 2011). However, an exceptional year occurred in 1980 when southwestern NSW had a “severe caterpillar plague” with *A. infusa* particularly prominent (Drake and Farrow, 1985). In that year the first arrivals in the Snowy Mountains were recorded on September 3rd (Ken Green, unpublished data). These first arrivals do not occupy the estivation sites as most are still choked with snow.

**Figure 8:**
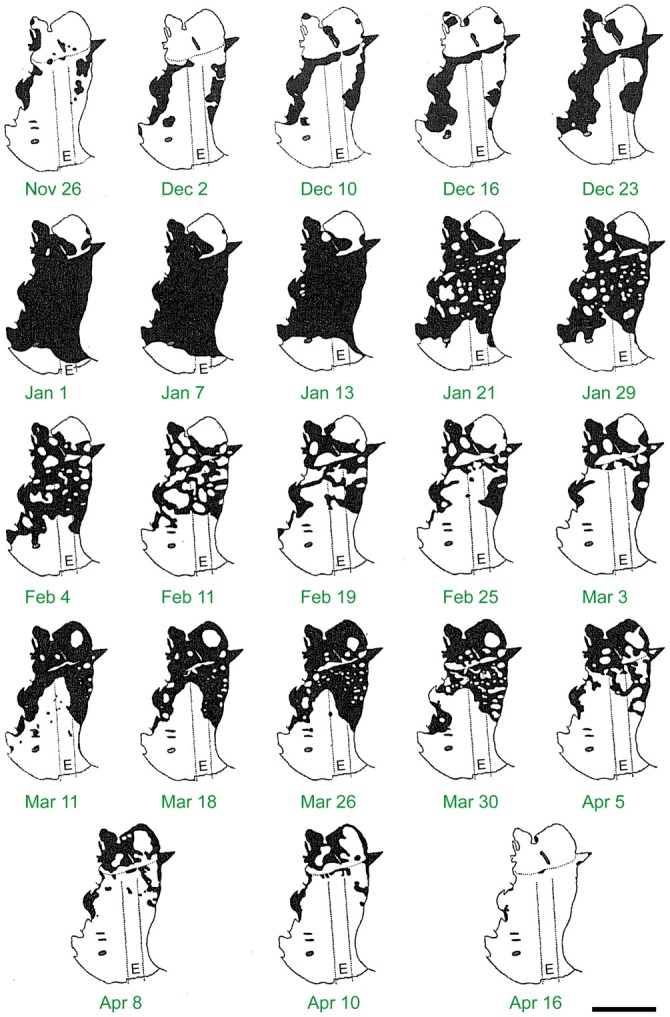
**The build up and decline of Bogong moth numbers on the roof of Ian Common’s observation cave on Mt Gingera over the summer of 1952–1953.** Each panel shows the area of the cave roof monitored and the *black areas* are the areas covered by Bogong moths. Numbers became maximal at New Year and then slowly declined until the beginning of March, with moths gradually disappearing first from the more exposed parts of the cave near its entrance *(E)*. Note the upswing in moth numbers from mid to late March—moths migrating from further south were apparently using this cave as a temporary shelter during their northward journey. *Dotted lines* show the width and length of the trench Common dug to assist entry to the cave. Scale bar = 2 m. Reproduced from Common (1954), with permission from CSIRO Publishing.

At the most northerly estivation site at Mt Gingera, Bogong moths start to occupy the caves from the end of October and increase to a maximum by mid-December (Figure 8; Common, 1954). However, most caves in the Snowy Mountains will usually have some snow at this time of year and may not initially be available—even caves buried in snow in early December are occupied later (Blakers, 1980). Thus, the peak number of Bogong moths at estivation sites in the Snowy Mountains occurs in January (Green, 2011). As the summer progresses, numbers at lower elevations drop off rapidly while they remain high at higher elevations (Green, 2010b).

Although Common (1954) was able to estimate the numbers on one wall of a cave (144,000 moths), obtaining a total count of moths arriving in the mountains is an intractable problem as in any rock complex the proportion of moths that is visible is an unknown proportion of the whole, and deeper, smaller cracks are the preferred sites (Green, 2010b). The numbers of moths flying to the mountain each year is in the billions, and at its peak in January, the number of estivating moths is likely to be in the order of two billion individuals (Green, 2010b). Of these, around half are likely to perish due to bad weather, predation and parasitism (Green, 2011).

The return migration begins from early February leading to a rapid decline in Bogong moths in estivation sites (Figure 8). However, Mt Gingera is also used as a temporary camp by Bogong moths on their northern migration from further south, leading to a second peak in numbers in late February to March (Figure 8; Common, 1954). At Mt Gingera, most moths have left by late March to early April (Common, 1954) and in the Snowy Mountains most have left by April, although some may stay until May (Green, 2011).

### Estivation

The spring generation of Bogong moth adults undergoes estivation (or summer diapause). This summer diapause is facultative and a small proportion of the spring migrants may breed if conditions are favorable, such as in suitable irrigated areas or in other areas encountered *en route*, such as gardens in Canberra (Common, 1954). If there were adequate larval food sources in the breeding areas, several generations of Bogong moths each year would theoretically be possible (Figure 5; Common, 1954). Because this is generally not the case, moths must therefore migrate. However, even within their alpine caves, where air temperatures average 9.8°C, moths would complete sexual development in less than 50 days, and some do reach maturity without estivating (Common, 1954). Indeed, some moths caught in light traps in Canberra in spring had already copulated, and larvae have been found in Canberra gardens in December (Common, 1954). Because of this, the best option to prolong sexual development is with facultative estivation (Common, 1954). This summer estivation helps delay the breeding season so that eggs do not hatch into an environment with poor food and high temperatures.

At estivation sites on Mt Gingera, Bogong moth numbers build from October and reach a maximum in December (Figure 8), with the first arrivals claiming the deepest, darkest sites (Common, 1954, 1981). These were crevices 50 mm in width, very dark and probably the most stable in temperature and humidity (Common, 1954). The moths hold onto the rock face with their fore tarsi, placing their hind legs on the backs of other moths lying beneath Common (1954). Moths joining the aggregation push themselves beneath the abdomen and wings of the moth in front so that their light-sensitive eyes are kept in the dark. In this formation, lined up side by side, each with its head stuck beneath the moth on the wall above it, they can achieve densities of 17,000 moths per square metre (Figure 3B; Common, 1954). This also enables them to retain body moisture, although it is worth noting that moths avoid wet rock surfaces (Common, 1954). As the aggregation grows, moths joining the formation may end up in less preferred locations. Indeed, some end up exposed to direct sunlight (Common, 1954), and these more peripherally located moths are possibly the first to be preyed upon. When disturbed, moths drop onto the cave floor, spreading from the center of the disturbance, but only for a short time before again seeking shelter (Common, 1954). These disturbed moths also “deposit drops of excrement” (Common, 1954), possibly as an anti-predator defence.

Moths can remain quiescent for several weeks, and possibly as long as 4 months (Common, 1954). After finding a small cave on Mt Gingera containing about 10,000 Bogong moths, Common prevented them from escaping from December until April by enclosing the cave entrance with wire mesh. He found that the moths remained quiescent for almost the entire time of confinement, only becoming (greatly) agitated in April when the main autumn migration was in progress (Common, 1952, 1954). At this time the moths flew towards the mesh and attempted to escape.

In cave populations studied by Common (1954) over 2 years, the sex ratio appears to be significantly male biased, with 58.5% and 61.5% of Bogong moths sexed as male. These ratios were significantly higher than ratios from moths reared from eggs, with 51.9% of moths being male (Common, 1954). By contrast, the fractions of male moths caught in spring, by high and low intensity light traps in Canberra, were 49.9% and 52.9%, respectively. When collecting moths from caves we have also found a strong male bias. It may be that females preferentially occupy the deeper narrow fissures within the caves that would be better refuges from predation and adverse weather.

#### Estivation Site Characteristics

Common (1954) used the term “camp” for both temporary camps and estivation sites. Estivation sites, being usually occupied in all years, are generally a location “where substantial moth debris occurs within a rocky environment” (Blakers, 1980). This debris, consisting of the decomposed bodies of moths in the bottom of the caves, was recorded as 30 cm deep at Mt Gingera (Common, 1954) and 128 cm deep in the Bogong Mountains (Vyner, in Scott, 1869), but in most caves debris does not accumulate to this depth due to the action of water washing it out. Bogong shelter #2, an estivation site at 1433 m in the ACT, has had its cave floor debris (including Aboriginal artifacts) dated to an age of approximately 1000 years (Flood, 1980, p. 246). Estivation sites and temporary camps above 1500 m, were mapped in the Snowy Mountains by Green (2010b), and these were generally complex rock tors or periglacial boulder fields and boulder streams (Green, 2010b). Blakers (1980) found an average density within his study area of 0.9 estivation sites per square kilometre.

In some years however, temporary camps at lower elevations may be used as estivation sites (Common, 1954). This use of temporary camps can extend into mid December (Common, 1954 Ken Green, personal observations) and moth flights throughout this period can consist of thousands of moths flying unidirectionally uphill (Ken Green, personal observations), which is quite distinct from the nightly random flights performed by low numbers of moths above the cave during their estivation as observed by Common on Mt Gingera (Common, 1954).

**Figure 9:**
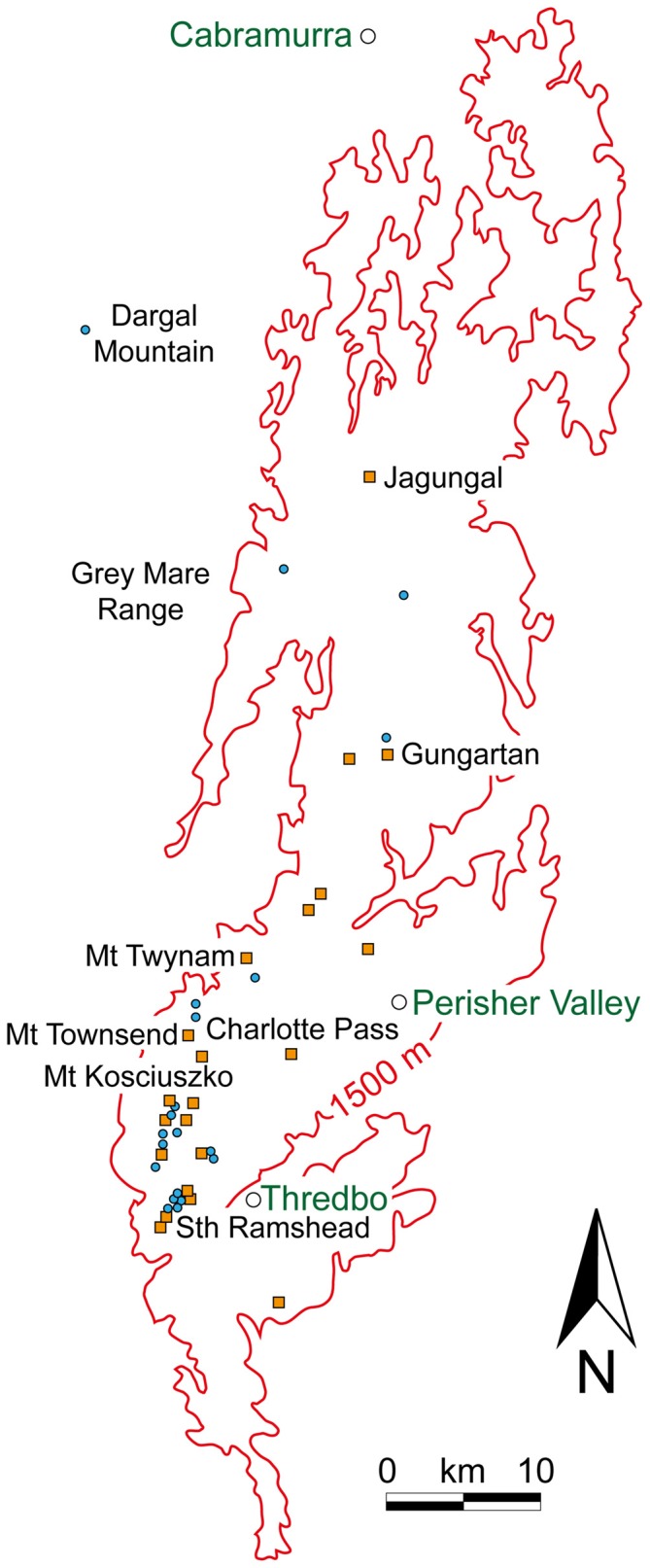
**The known estivation sites *(orange squares)* and temporary camps *(blue circles)* for Bogong moths above 1500 m *(red isoline)* in the Snowy Mountains of NSW.** The geographical region represented in this figure is the region bounded by the *white box* in Figure 6. Town names are shown in *green text*. Redrawn from Green (2010b).

As mentioned above, the most northerly known estivation site occurs at Mt Gingera on the ACT/NSW border with sites to the west in the Bogong Peaks and to the east in the Tinderry Range (see Figure 6 for the locations of some peaks mentioned below). The major sites however are further to the south in the Snowy Mountains (Figure 9), with an isolated site on Mt Morgan (1852 m) at the northern limit of these mountains. Most sites are concentrated from Mt Jagungal (2061 m) in the north to the Chimneys (1885 m), just south of Thredbo. Further south on the NSW/Victorian border another isolated site occurs on Mt Tingaringy (1448 m) and within Victoria sites occur at Mt Cobberas (1810 m), Mt Bogong (1986 m), Mt Cope (1837 m), Mt Hotham (1862 m) and further to the west on the isolated Mt Buffalo (1723 m). The highest estivation site is on Australia’s highest mountain, Mt Kosciuszko (2228 m), and the lowest elevation at which estivation is thought to occur on the mainland is at 1200 m, below the summit of Mt Tingaringy (Green, 2010b). In the ACT the lower known limit for estivation sites occurs at approximately 1350 m (Flood, 1980). Tasmania is a special case, where migration to the island and subsequent estivation is not a strictly annual affair. Nonetheless, moths will estivate in locations such as the scree slopes on Mt Barrow (*ca*. 1400 m) and possibly several other sites in Tasmania, returning to the mainland in autumn (Hill, 2007).

#### The Internal Climate of Estivation Caves

Due to the general air circulation, air flowing through crevices in tors and boulder fields gives the caves a dynamic atmosphere, with internal temperatures almost immediately following those of the outside air (Geiger, 1965). Temperatures inside caves are reasonably close (0.3–0.5°C) to those outside within nearby meteorological screens (Green, 2010b). Similarly, relative humidity in each cave follows changes in external humidity, but is always about 10% higher in estivation sites (Green, 2010b). Humidity could be somewhat higher where Bogong moths congregate, restricting loss of moisture to the general cave air (Common, 1954). Protection from wind is almost complete in estivation sites and all sites are protected from direct precipitation (Blakers, 1980). However, this does not prevent moths being washed out of caves and killed in downpours. Blakers (1980) reported thousands of dying moths due to flooding of a poorly drained site and Green et al. (2001) reported an outwash of dead moths from a number of estivation sites after very heavy rains.

Common (1954) commented on the westerly aspect of most sites around Mt Gingera but suggested that local geomorphology may play a part. However, Blakers (1980) found a distinct southerly aspect in estivation sites around Mt Townsend (2209 m) in the Snowy Mountains but discounted local geomorphology as the cause, since suitable sites were found with all aspects. In contrast, an examination of the aspects of 92 temporary camps and 42 estivation sites, over a much wider geographical area, found no significant preference (Green, 2010b). On Mt Barrow (1414 m), one of the only places where moths are known to estivate in Tasmania, the scree slope used has a northeasterly aspect (Hill, 2007).

#### Moth Activity During Estivation

Once moths enter diapause they show very little activity and the accumulated pattern of moths on the cave walls changes little through the season (Common, 1954). A small proportion of the moths do, however, participate in random flights above the cave a little after sunset (for about 1 h: Common, 1954), and before commencing these evening flights they vibrate their wings to warm the wing muscles. As the moths fly, there is a deep humming sound (Common, 1981). Kevan and Kendall (1997) suggested that Bogong moths might undertake nightly summer nectarivory. This was not the case on flights recorded by Common (1954), which did not include feeding, even when snow gums were in full flower at the top of Mount Gingera (I. F. B. Common, 2005, personal communication). It may, however include drinking (Blakers, 1980; Common, 1981; Ken Green, personal observations). No breeding occurs either at arrival at the estivation sites, nor on departure, and larvae cannot be found close to the estivation sites (Common, 1952).

#### Predation and Parasitism During Estivation

The main predators of estivating Bogong moths are Little ravens, Bush rats, Richard’s pipits and Red foxes (Green, 2003, 2011). Based on published and estimated densities of these and other known predators of Bogong moths, Green (2011) calculated the intake of Bogong moths as food. Adding the death toll from parasitism and adverse weather to the death toll through predation, the calculated mortality for the entire Snowy Mountains is estimated to be greater than one billion moths annually. This mortality contributes seven tonnes of Nitrogen, one tonne of Phosphorus and over 5000 Gigajoules of energy annually (Green, 2011).

Bogong moths are parasitized by two species of mermithid nematodes, *Amphimermis bogongae* and *Hexamermis cavicola* (Welch, 1963). Before the moths arrive in the cave, the early instar nematode larvae leave the detritus of the cave floor (where they hatched from eggs), to swim upwards in water channels trickling down the cave walls. After the moths arrive, these larvae enter their hosts, either via the proboscis while the moths are drinking from the water trickles, or via their spiracles as shown experimentally in the moths *Phryganidia californica*, and *Galleria mellonella* (Triggiani and Poinar, 1976). The nematode larvae leave the moth’s body in late January to early February (Common, 1990), killing the moth in the process. The free-living larvae, up to 19 cm long, then return to the debris of the cave floor where they spend the winter burrowed to a depth of 20–35 cm. Here they mature, mate and lay their eggs (Common, 1981). The dependence of the parasites on the return of the moths in spring highlights the long-term association of Bogong moths with particular estivation sites.

## The Sensory Basis of the Bogong Moth Migration

The ability of animals as varied as birds, sea turtles and insects to migrate vast distances, over land or through the ocean, and to navigate successfully to specific destinations, has fascinated biologists for decades. What has become clear from years of research is that migrating animals rely heavily on a wide range of sensory cues to achieve this (Frost and Mouritsen, 2006), with different types of sensory information being used during three distinct phases of migration (Mouritsen et al., 2016): (1) a long distance orientation phase in which animals use global compass cues (e.g., celestial and/or geomagnetic cues) to migrate in particular directions, and over large distances, from their geographic origin to arrive in the vicinity of their destination; (2) a narrowing-in phase in which animals use a range of local sensory cues within the vicinity of their destination (including landmarks, and sensory gradients) that bring them closer their final goal; and (3) a pinpointing phase in which animals use destination-specific sensory cues (such as specific landmarks) to locate their final goal.

For instance, magnetic cues and/or celestial visual cues (the sun, moon, stars and celestial polarized light) can be used as a compass to choose (and hold) a desired course (migratory phase 1), while other sensory cues (e.g., magnetic and olfactory cues, and visual landmarks) can be used to determine when a destination has been reached (migratory phases 2 and 3). Some animals—such as adult birds (Kishkinev et al., 2013, 2015), fish (Putman et al., 2014), lobsters (Boles and Lohmann, 2003) and sea turtles (Brothers and Lohmann, 2015)—also seem in addition to have a map sense used to determine their geographic location on the Earth’s surface based on a combination of different cues, which can include olfactory cues (Gagliardo, 2013), the angle of the sun or celestial center of rotation above the horizon (Kramer, 1953; Mouritsen, 2003), and/or the local inclination and intensity of the Earth’s magnetic field, a natural mechanism likened to a modern GPS navigation system (Lohmann et al., 2007). These animals are thus said to be capable of “true navigation” (Kramer, 1953; Mouritsen, 2003). Whether insects have this ability is hotly debated (see Cheeseman et al., 2014; Cheung et al., 2014), but recent evidence suggests that in the Monarch butterfly at least, a map sense and true navigation does not exist (Mouritsen et al., 2013). However, even without a map sense, Monarch butterflies accurately locate their migratory destinations, and are likely to use a number of sensory cues—including the disc of the sun (Mouritsen and Frost, 2002; Froy et al., 2003; Heinze and Reppert, 2011) and a funneling effect created by geographical landmark barriers (Mouritsen et al., 2013)—to steer their migratory flight and arrive safely in Mexico. One study has also suggested that Monarch butterflies use the celestial pattern of polarized skylight as a compass cue during navigation (Reppert et al., 2004), although a later study found that if used at all, polarized light is a minor cue compared to the disk of the sun (Stalleicken et al., 2005). Finally, even though there are now reports suggesting that Monarch butterflies can sense magnetic fields (Guerra et al., 2014), it is still an open question whether they use the Earth’s magnetic field—in addition to the sun’s disc—as a compass to steer their migratory flight in natural circumstances (Mouritsen and Frost, 2002; Stalleicken et al., 2005).

The Bogong moth must also rely on sensory cues for its nocturnal migration. The moon’s disk, and the polarization pattern produced in the sky around it, are obvious compass cues for use during the first migratory phase. So too are the stars. Indeed, nocturnal dung beetles are able to use all three cues when rolling balls along straight trajectories away from the competitive fury of the dung heap (Dacke et al., 2003, 2004, 2013), with these cues arranged in a hierarchy of descending importance (polarized light, moon’s disk, and then stars). However unlike Bogong moths, which must hold a compass direction all night, dung beetles seldom maintain their straight-line rolling for more than a few tens of minutes at most and during this time nocturnal celestial cues remain fairly invariant. In contrast, over an entire night, the rotations of the Earth and the moon cause dramatic changes in the positions of celestial cues, and their usefulness as a compass for an all-night navigator diminishes. Moreover, unlike the sun that has an almost constant trajectory across the sky day after day, the moon is a more fickle cue, changing in both its presence and prominence over the lunar month (with consequences for the presence and intensity of its associated polarization pattern). For a Monarch butterfly that migrates throughout the day, the predictable position of the sun at any given time relative to the butterfly’s desired migratory direction can be compensated for as the sun moves across the sky (Mouritsen and Frost, 2002; Froy et al., 2003). Even though a time-compensated moon compass has been demonstrated in the short-range beach migrations of sandhoppers to and from the waterline (Ugolini et al., 2003), for a long-distance nocturnal navigator such as the Bogong moth the moon is a less reliable cue than the sun is for a diurnal navigator. Not surprisingly, other nocturnal navigators, such as birds, have turned to the Earth’s magnetic field as a reliable compass cue (Wiltschko and Wiltschko, 1972; Cochran et al., 2004), and it may well be the case that Bogong moths (and other nocturnal long-distance insect navigators) have done the same.

### How Might Sensory Information be Used During the Migratory Flights of Nocturnal Moths?

Like the Bogong moth, many nocturnal insect species (particularly moths) undertake spectacular long-distance seasonal migrations at specific times of the year, moving in a common direction at high-altitudes to exploit temporary breeding habitats (Riley and Reynolds, 1986; Riley, 1989; Gatehouse, 1997; Chapman et al., 2015b) or to re-locate to suitable hibernation or estivation sites (Rainey, 1951; Dingle, 1996), some of which can be rather restricted in area (a few hundred square kilometres or less).

These nocturnal migrations (which are characteristic of the first migratory phase) frequently take advantage of favorable fast-moving winds (Riley et al., 1983, 1995; Drake, 1985; Feng et al., 2005; Chapman et al., 2008a,b), and can take migrating insects hundreds of kilometres in one night (Drake and Farrow, 1985; Chapman et al., 2008a). Indeed, in a vertical radar study by Drake and Farrow (1985), the migratory directions of various species of moths, including Bogong moths close to their breeding grounds (and flying at an altitude of up to 1 km), were basically downwind. But far from being completely at the mercy of the prevailing winds, nocturnal insect migrants have recently been shown to have the ability to align their flight directions relative to the wind direction (Schaefer, 1976; Riley and Reynolds, 1986; Chapman et al., 2008a,b, 2010; Reynolds et al., 2010). In fact Ian Common had already noted this for Bogong moths in the early 1950’s—by carefully measuring the flight directions and numbers of moths (flying a few metres above the ground) that were arriving over several nights at Mt Gingera during the spring migration, and then again when leaving during the autumn migration, he found that the spring moths were steadily aligned south to southwest, while autumn moths were steadily aligned north to northeast, despite wind directions being much more variable (Common, 1954, Tables 1 and 2). Our own measurements of Bogong moths flying in a wind-free flight simulator (of the type used by Mouritsen and Frost, 2002) agree with Common’s observations—migrating moths captured in spring on Mt. Kaputar in northern NSW are predominantly oriented southwards, while migrating moths captured in autumn in the Snowy Mountains are predominantly oriented northwards (personal observations). Moreover, Green (2006) observed a dense stream of Bogong moths during the autumn migration, flying a few metres above the ground near Mt Jagungal (see Figure 9), that were returning northward despite a steady NNW headwind of up to 14.5 km/h (and on this evening these winds remained in this direction to an altitude of 4000 m).

How do moths align their flight in other directions than that dictated by the wind? Recent work in moths shows that they are able to detect and respond to wind turbulence and to compensate partially for crosswind drift, thereby significantly increasing their migratory distance (Chapman et al., 2010, 2015a; Reynolds et al., 2010). Another possibility is that moths assess the visual optic flow of landscape features on the ground below to compensate for this drift. Optic flow cues are highly important for the control of flight in insects (Srinivasan et al., 2006), including moths (Vickers and Baker, 1994), and it is not unreasonable to imagine that ground-based optic flow could be used to maintain a desired flight direction. However, irrespective of the cues used to compensate for crosswind drift, to achieve a common orientation at high altitude, migratory moths need to have a “preferred inherited direction”—as Bogong moths evidently do—and to maintain this direction throughout the night. Thus, they ultimately need to rely on some form of compass, but the nature of this compass remains enigmatic.

Three main celestial visual cues could be used as a nocturnal compass for long-distance navigation: the disk of the moon, the dim pattern of polarized light formed around the moon and the constellations of stars. As mentioned above, most of these cues have the disadvantage of altering their celestial positions throughout the night (requiring a time compensation mechanism for accurate navigation), and lunar cues (unlike solar cues) also vary in their duration and prominence at different times of the month. However, if Bogong moths can detect individual prominent stars and learn to determine the celestial center of rotation from these, as birds are known to do (Emlen, 1975; Wiltschko et al., 1987; Mouritsen and Larsen, 2001), a very robust, time-independent star compass can be established and used.

Many moths are able to orient for long periods of time along a chosen bearing even on clear moonless nights, and radar recordings of mass migration events have revealed a common orientation direction when the moon is well below the horizon (Riley and Reynolds, 1986; Chapman et al., 2008a), suggesting the possibility of a stellar compass (Sotthibandhu and Baker, 1979), although the evidence for this is still tenuous. Depending on the sensitivity of the eye (and it is currently unknown how many stars would be visible to the small compound eyes of the Bogong moth), the insect could potentially orient to a single lodestar (Doujak, 1985; Mauck et al., 2008) or identify the center of celestial rotation as birds do. Moreover, the center of celestial rotation (which always indicates geographic South in Australia) may also influence migratory direction, as has been demonstrated in North American passerines (Emlen, 1970). Alternatively, as has been determined for dung beetles, Bogong moths might be able to orient relative to the bright stripe of the Milky Way, a much more prominent visual cue than a single star (Dacke et al., 2013). However, the passage of the Milky Way across the sky from dusk to dawn would require moths to time compensate this potential cue during their nightlong migrations.

Interestingly, Bogong moths maintain their preferred migratory heading even on completely overcast nights when all visual celestial compass cues are obscured (Common, 1954, Tables 1 and 2; personal observations), suggesting that Bogong moths might possess a magnetic compass sense for holding their migratory direction, as found in many species of nocturnal migratory birds. However evidence for this possibility is still lacking, both for Bogong moths and other species of nocturnal moths.

Another possibility, at least for Bogong moths flying between the Australian Alps and northern NSW and southeastern Queensland, is that global compass cues are combined with a distinct geographical cue—in this case the long and continuous ridge of the Great Dividing Range (that runs just inland along the entire east coast of Australia, see Figure 6). If migrating moths could keep to the highest points along this ridge—using visual, thermal, barometric and/or other sensory cues—then this ability would bring them eventually to the snow-capped peaks of the Australian Alps in spring, and away from them in autumn. Such geographic “funneling” seems to be a feature of the Monarch butterfly migration (Mouritsen et al., 2013).

### How Might Sensory Information be Used to Identify the Migratory Destination?

Even though a critically important aspect of migration is to use compass information to identify and hold a desired migratory direction, an equally important migratory task is to determine when and/or where the journey should end (migratory phases 2 and 3). In the case of a Bogong moth, this requires the identification of the estivation site, one of the several isolated caves or crevices that dot the high alpine ridges of the Australian Alps and which have been used by moths for thousands of generations (Figure 9). How might this be achieved?

Several possible sensory cues are available, none of which however have yet been investigated. Visual cues are an obvious possibility. For instance, at the time of year when the moths arrive the alpine peaks are still covered in snow, and even at night the snowfields would create a bright visual beacon unique to this part of the Great Dividing Range, especially if lit by the moon or stars. Certainly the superposition compound eyes of the Bogong moth, an eye design exquisitely sensitive to dim extended scenes (Warrant, 2004, 2006; Warrant and Dacke, 2010, 2011, 2016), would have no difficulty discerning such cues at night. But because the moths begin their time in the mountains at lower elevations, and only gradually make their way upwards to the highest peaks before finally entering a cave, if these possible snow beacons are used at all they are likely to function as a general cue for narrowing in on the mountains and ceasing migration, rather than allowing identification of a specific estivation site. Other sensory cues a Bogong moth could potentially use to signal its approaching alpine destination (also as general cues to cease migration) is barometric pressure and temperature, both of which decline with increasing elevation.

How then might the final specific estivation site, such as a cave, be located? An obvious sensory cue is the odor of the cave itself. As mentioned above, the cave floor debris consists of the remains of dead bodies and excrement from countless generations of moths from previous years, and even to our own comparatively insensitive olfactory system, the smell of this debris (and thus the smell of the cave) is distinct and pungent. For a Bogong moth—with an exquisitely sensitive sense of smell typical of moths—the cave odor could easily act as an olfactory beacon when the moth gets within range. So too could any type of aggregation pheromone or other excretion produced by moths already on site. Olfactory beacons have also been suggested as a possible cue used by migrating Monarch butterflies to locate their final roosts (Frost et al., 2008; Reppert et al., 2010; Mouritsen et al., 2013), the isolated Oyamel fir forests of central Mexico.

Another possibility is that Bogong moths rely on a magnetic map sense (probably in combination with other cues) to either narrow in on their estivation sites and/or to pinpoint their final destination precisely. However, evidence is lacking for such a map in insects, including the Monarch butterfly (Mouritsen et al., 2013), despite the fact that these insects might clearly benefit from such a map. Interestingly, a magnetic map sense does exist in another arthropod, the Spiny lobster (Boles and Lohmann, 2003). It could be argued that migratory insects such as Monarch butterflies and Bogong moths have sufficient sensory cues to recognize their final destinations without the need for a magnetic map (and this might well be the case), but exactly how this important part of the migratory journey is achieved remains an open question.

## Conclusions

Like the North American Monarch butterfly, the Australian Bogong moth *Agrotis infusa* is an iconic long-distance navigator. Its remarkable yearly return journey from the hot plains of southeastern Australia to isolated cool caves along the ridgetops of the Australian Alps, its occasional disruption of the business of government in the national capital Canberra and its folkloric importance for the Australian Aborigines, have all entrenched the Bogong moth in the psyche of the Australian people. Moreover, their predictable presence in the mountains each summer is a major factor in the health and wellbeing of the entire alpine ecosystem. Without doubt, the Bogong moth is one of Australia’s most extraordinary and best-known insects.

While lacking the visual splendour of the brilliantly colored Monarch butterfly, the small and humbly decorated Bogong moth is equally impressive in terms of its navigational skills. The navigational problems that both Lepidopterans need to solve as they fly towards, and then arrive, at their migratory destinations are very similar. However, the Monarch butterfly solves these problems during the day using the bright and reliable disk of the sun as a compass for finding its way south, while the Bogong moth must solve them at night when visual compass cues are much less reliable and/or much more difficult to detect—the variable presence and prominence of the moon (and its associated polarization pattern) over the lunar month, as well as the nightly movement of the stars, all degrade the reliability of these cues for navigation throughout the night. Exactly how the Bogong moth nonetheless finds its way to the Australian Alps—and then identifies its final estivation site—is still an open and very interesting question. One intriguing possibility is that like nocturnally migrating birds, the Bogong moth relies on compass cues from the Earth’s magnetic field to steer its journey in the right direction. The identification of the sensory mechanisms used by Bogong moths during their migration is a fascinating and potentially fruitful area for future research.

## Author Contributions

Conception: EW, BF, KG and SH. Text and/or figures: EW, BF, KG, HM, DD, AA, KB and SH. Critical review of the manuscript: EW, BF, KG, HM, DD, AA, KB and SH.

## Conflict of Interest Statement

The authors declare that the research was conducted in the absence of any commercial or financial relationships that could be construed as a potential conflict of interest.
