# Corrigendum: The Australian Bogong Moth *Agrotis infusa*: A Long-Distance Nocturnal Navigator

**DOI:** 10.3389/fnbeh.2017.00162

**Published:** 2017-08-30

**Authors:** Eric Warrant, Barrie Frost, Ken Green, Henrik Mouritsen, David Dreyer, Andrea Adden, Kristina Brauburger, Stanley Heinze

**Affiliations:** ^1^Lund Vision Group, Department of Biology, University of Lund Lund, Sweden; ^2^Department of Psychology, Queens University Kingston, ON, Canada; ^3^New South Wales National Parks and Wildlife Service Jindabyne, NSW, Australia; ^4^Institute for Biology and Environmental Sciences, University of Oldenburg Oldenburg, Germany

**Keywords:** Bogong moth, *Agrotis infusa*, insect, migration, navigation, estivation, vision, magnetoreception

In the original article, there was a mistake in the legend for Figure 4 as published. We were unaware that the map of Australian Aboriginal tribal boundaries used in this figure, despite acknowledgment of the source (Tindale, 1974), was still under copyright to Tony Tindale and Beryl George (administered by the South Australian Museum). Use of this map requires the permission of the South Australian Museum and a disclaimer concerning the map itself. The correct legend is as follows:

FIGURE 4 | The traditional Aboriginal tribal boundaries of southeastern Australia, and a nineteenth century portrait of an Aboriginal man from the Monaro district of the Snowy Mountains wearing the apron-like bridda bridda. The map is a reproduction of N. B. Tindale's 1974 map of indigenous group boundaries existing at the time of first European settlement in Australia (Tindale, 1974). It is not intended to represent contemporary relationships to land. © Tony Tindale and Beryl George, 1974. Portrait: Photo no. 1304 by Henry King (1855–1923), from the Tyrrell Collection (7903 glass plate negatives from the studios of Henry King and Charles Kerry (1858–1928), held at the Powerhouse Museum, Sydney, and available through the Commons on Flickr).

The authors apologize for this error and state that this does not change the scientific conclusions of the article in any way.

The original article has been updated.

## Conflict of interest statement

The authors declare that the research was conducted in the absence of any commercial or financial relationships that could be construed as a potential conflict of interest.
